# Modeling and analysis of cell membrane systems with probabilistic model checking

**DOI:** 10.1186/1471-2164-12-S4-S14

**Published:** 2011-12-22

**Authors:** Mirlaine A Crepalde, Alessandra C Faria-Campos, Sérgio VA Campos

**Affiliations:** 1 Department of Computer Science, Federal University of Minas Gerais, Av. Antônio Carlos 6627, Belo Horizonte, MG, Brazil

## Abstract

**Background:**

Recently there has been a growing interest in the application of Probabilistic Model Checking (PMC) for the formal specification of biological systems. PMC is able to exhaustively explore all states of a stochastic model and can provide valuable insight into its behavior which are more difficult to see using only traditional methods for system analysis such as deterministic and stochastic simulation. In this work we propose a stochastic modeling for the description and analysis of sodium-potassium exchange pump. The sodium-potassium pump is a membrane transport system presents in all animal cell and capable of moving sodium and potassium ions against their concentration gradient.

**Results:**

We present a quantitative formal specification of the pump mechanism in the PRISM language, taking into consideration a discrete chemistry approach and the Law of Mass Action aspects. We also present an analysis of the system using quantitative properties in order to verify the pump reversibility and understand the pump behavior using trend labels for the transition rates of the pump reactions.

**Conclusions:**

Probabilistic model checking can be used along with other well established approaches such as simulation and differential equations to better understand pump behavior. Using PMC we can determine if specific events happen such as *the potassium outside the cell ends in all model traces*. We can also have a more detailed perspective on its behavior such as determining its reversibility and why its normal operation becomes slow over time. This knowledge can be used to direct experimental research and make it more efficient, leading to faster and more accurate scientific discoveries.

## Background

Computational modeling has been increasingly used in the field of systems biology to examine the dynamics of biological processes. Traditionally, the modeling of biochemical pathways is based on a set of non-linear ordinary differential equations (ODE) to describe the evolution of average molecular concentrations over time [1]. This approach assumes continuously varying chemical concentration and deterministic dynamics, which can be unsuitable for some classes of systems, such as those that need stochastic modeling or contain a small number of molecules for each species.

The main alternative modeling paradigm, originally proposed by Gillespie [2], focus on stochastic models. It produces counts of molecules of some chemical species, whose rates of interaction are controlled by exponential distributions. In a stochastic model, various possibilities exist for the future behavior of the system, where each possibility has a certain probability. The usual way of analyzing those models is via simulation, producing trajectories that provide us with different insights of the system. Therefore, if we want to use simulation to recover meaningful information about the behavior of the system we often need a large number of simulations runs in order to retrieve an accurate estimation.

Recently there has been considerable interest in the application of model checking [3] as a powerful tool for formally reasoning about the dynamic properties of biological systems (e.g. [4], [5], [6]). Model Checking provides a way to both formally describe and analyze a system. It is a well-established and widely-used formal method for ascertaining the correctness of real-life systems. This approach is able to explore all behaviors of a modeled system through an exhaustive and systematic exploration of all possible states of the system, and therefore can identify events and conditions that can be overlooked by simulation. Probabilistic Model Checking, or PMC, is a variant of model checking for modeling and analyzing systems that exhibit stochastic behavior as is the case for several biological systems. Similar to stochastic simulation, PMC is based on a stochastic and discrete-state modeling approach via Continuous Time Markov Chains (CTMCs). However, the output of PMC is exact, as opposed to the output of stochastic simulation which is inherently approximated, taking averages over sets of simulation runs. Moreover, given that PMC deals with all states of the system, it is possible to precisely verify if an observation (a property of interest or an event) will continue forever or rather will definitely stop.

We propose that PMC be used in addition to stochastic and deterministic simulation in order to amplify the understanding of the biological system. For example, PMC can give clues about the existence of some events that can be later checked with stochastic simulation through the recovery of traces where the specific event happens. It also can support biologists suggesting interesting but uncommon aspects that can be verified experimentally.

In this paper we will use PMC for the modeling and analysis of the sodium-potassium exchange pump (Na,K-pump) in a quantitative way. This pump is an important transport system present in all animal cell and responsible for keeping the potassium and sodium concentrations inside the cell, respectively, high and low. Low sodium concentrations and high potassium concentrations in the cell cytoplasm are essential for basic cellular functions such as excitability, secondary active transport and volume regulation. In the brain, about one-half of the Adenosine Tri-Phosphate (ATP) provided by oxidate metabolism is used to power the Na,K-pump [7].

A formal specification of this system has already been developed using the *π* — *calculus* process algebra based on the known Albers-Post model [4]. This work has also used model checking to verify some computational properties such as deadlock and bisimilarity, which is an equivalence relation between state transition systems, associating systems which behave in the same way in the sense that one system simulates the other and vice-versa. However, it does not have a quantitative description of the Na,K-pump, nor does it deal with quantitative properties about the biological system.

We will describe how the pump mechanism can be modeled using probabilistic model checking taking into consideration a discrete chemistry approach and the Law of Mass Action aspects. We also will present some significative properties about the pump reversibility that can be addressed directly with model checking, whereas with other traditional approaches, such as deterministic and stochastic simulation, they can not be easily covered. Finally, we will reason about the pump behavior in terms of trend labels for the transition rates of the pump reactions which compute if there is a greater probability that the system takes specific transitions. These trends allow us to identify, for example, why the Na,K-pump goes more slowly in the forward direction over time, justifying the long periods of time to exhibit its reversibility.

## Methods

### Sodium-potassium exchange pump

**Figure 1:**
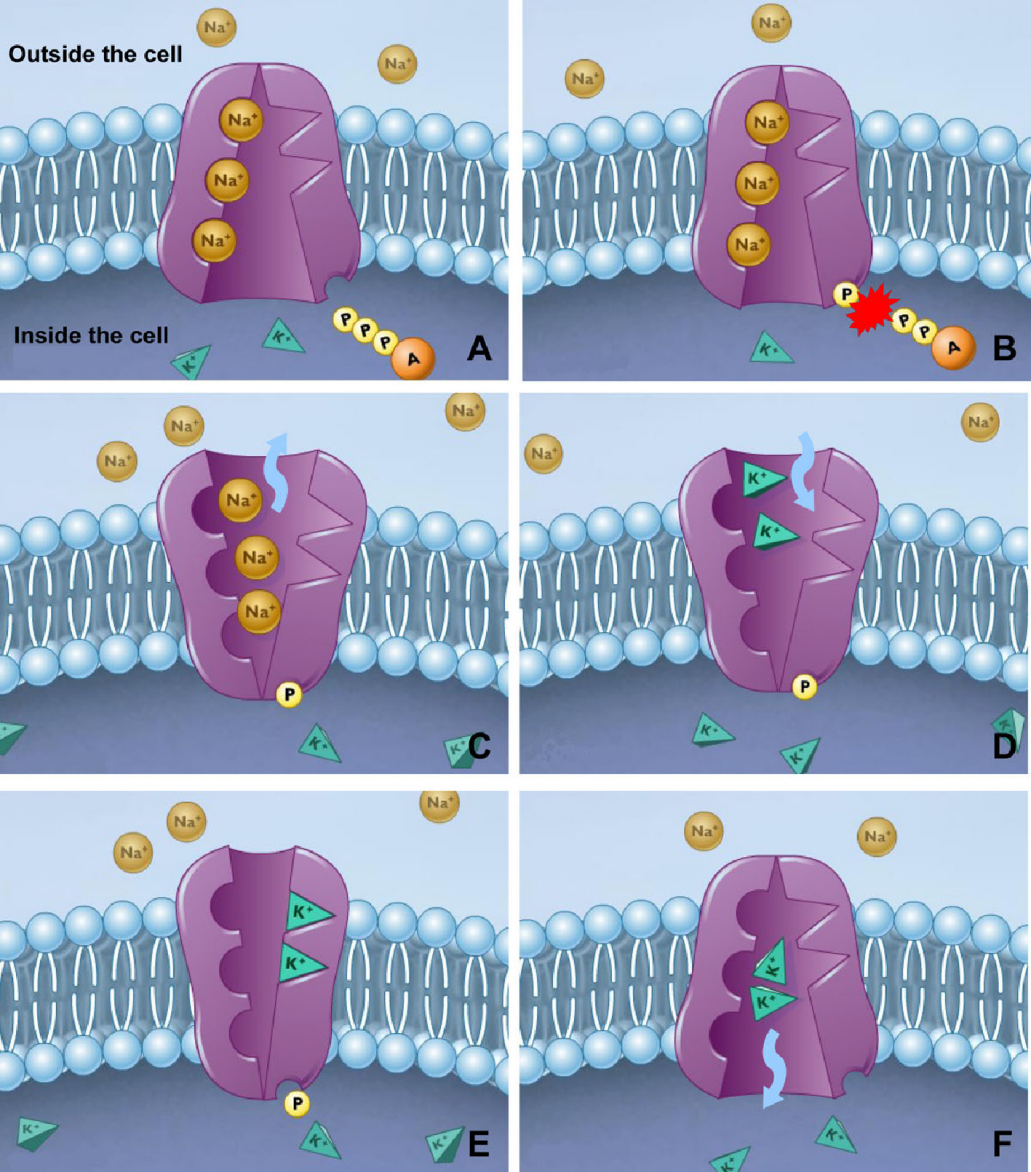
**The sodium-potassium exchange pump mechanism.** The Na,K-pump that moves two potassium ions from outside the cell to inside and three sodium ions from inside the cell to outside by the breakdown of ATP molecules.

**Figure 2:**
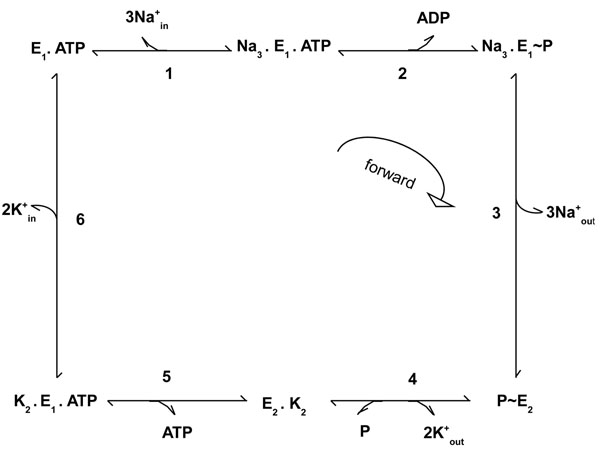
**Reaction scheme for sodium-potassium pump.** Reaction scheme for sodium-potassium pump mechanism based on Albers-Post model. *E*_1_ and *E*_2_ refer to conformationally distinct form of the pump, *P* is phosphate, *ATP* and *ADP* are adenosine tri- and di-phosphate respectively; , , ,  refer to intracellular and extracellular *Na*^+^ and *K*^+^, respectively.

The sodium-potassium exchange pump is found in the plasma membrane of virtually all animal cells and is responsible for the active transport of sodium and potassium across the membrane. One important characteristic of this pump is that both sodium and potassium ions are moving from areas of low concentration to high concentration, i.e., each ion is moving against its concentration gradient. This type of movement can only be achieved using the energy from the hydrolysis of ATP molecules. Figure 1 shows the Na,P-pump mechanism, which driven by a cell membrane ATPase, moves two potassium ions from outside the cell (low potassium concentration) to inside the cell (high potassium concentration) and three sodium ions from inside the cell (low sodium concentration) to outside the cell (high sodium concentration). Our modeling is based on the reaction scheme shown in Fig. 2 (quoted from [8]), which provides a summary of the Albert-Post cycle [9]. According to this cycle, the pump protein can assume two main conformations, *E*_1_ and *E*_2_, with inward-facing (*E*_1_) and outward-facing (*E*_2_) binding sites for sodium ions (Na^+^ ) and potassium ions (K^+^), respectively. The intracellular and extracellular forms of Na^+^ and K^+^ ions are explicitly identified as , ,  and . P_i_ is the inorganic phosphate group and *f_i_* and *b_i_* are the forward and reverse rate coefficients for the *i*^th^ step in the cycle. For example, *f*_1_ is the forward rate for the first step reaction . Moreover, A. B means that A and B are bound to each other noncovalently and *E*_i_ ~ P indicates that the phospharyl group is covalently bound to *E*_i_. The pump mechanism is decomposed into a set of six elementary and reversible reactions. The enzyme in its conformation *E*_1_ and with ATP already bound binds to three sodium ions inside the cell (step 1). This reaction stimulates ATP hydrolysis and then the release of Adenosine D—Phosphate (ADP) inside the cell, forming a phosphorylated enzyme intermediate (step 2). Extrusion of Na^+^ ions is completed by a conformational change (*E*_2_) and dissociation of the resulting complex (step 3). In this new shape, the pump has high affinity with potassium ions. Then, two potassium ions outside the cell bind to the pump enzyme and because of this reaction the enzyme is dephosphorylated (step 4). A further conformational change in which the enzyme binds ATP (step 5) is followed by the release of the two potassium ions inside the cell (step 6). Finally, the pump enzyme restores its original form that is capable of reacting with  at step 1. The quantitative data associated with this mechanism, quoted from [8] and [10], can be found in Table 1, which gives us a starting point for exploring the pump behavior in a quantitative way.

**Table 1:** Experimental data associated with the sodium-potassium pump cycle

Parameter	Value	Units
[]	0.02200	M
[]	0.14000	M
[]	0.12700	M
[]	0.01000	M
[ATP]	0.00500	M
[*P_i_*]	0.00495	M
[*ADP*]	0.00006	M
*f*_1_	2.5 × 10^11^	M^–3^s^–1^
*f*_2_	10^4^	s^–1^
*f*_3_	172	s^–1^
*f*_4_	1.5 × 10^7^	M^–2^s^–1^
*f*_5_	2 × 10^6^	M^–1^s^–1^
*f*_6_	1.15 × 10^4^	s^–1^
*b*_1_	10^5^	s^–1^
*b*_2_	10^5^	M^–1^s^–1^
*b*_3_	1.72 × 10^4^	M^–3^s^–1^
*b*_4_	2 × 10^5^	M^–1^s^–1^
*b*_5_	30	s^–1^
*b*_6_	6 × 10^8^	M^–2^s^–1^
cell volume	10^–12^	l
temperature	310	K

Normal physiological parameters associated with the scheme in Fig. 2.[], [], [], [], [*ATP*], [*P_i_*] and [*ADP*] are the concentrations of substrates. *f_i_* and *b_i_* are the rate constants, respectively, in the forward and backward direction for the reaction *i* in Fig. 2.

### Probabilistic model checking

Suppose *M* is a stochastic model over a set of states *S*, *s*_0_ is a starting state, *ϕ* is a dynamic property expressed as a formula in temporal logic, and *θ* ∈ [0, 1] is a probability threshold. The Probabilistic Model Checking [5,11] (PMC) problem is: given the 4-tuple (*M*, *s*_0_, *ϕ*, *θ*), algorithmically decide whether *M*, *s*_0_ |= *P*_≥*θ*_(*ϕ*), i.e. if the property *ϕ* is true with probability greater or equal than *θ*. In other words, probabilistic model checking requires, like non-probabilistic model checking, two key inputs: a description of the system in some high-level modeling formalism and a specification of one or more desired properties (*ϕ*) of that system in temporal logic. However, unlike non-probabilistic version, in probabilistic model checking the model is stochastic and the properties of interest are expressed in a quantitative way: for example, rather than verifying that ”does the species A eventually react with the species B?” we are interested in asking ”what is the probability that the species A eventually reacts with the species B?”. Given the stochastic description of the model, the probabilistic model checker constructs a mathematical model *M* that represents the system dynamics usually in terms of a digraph, in which each state represents a possible configuration and each transition represents an evolution of the system from one configuration to another over time. Moreover positive and real values are assigned to the transitions between states, representing rates of negative exponential distributions. This mathematical model *M* is, in fact, a *continuous-time Markov chains* (CTMCs) [5]. Formally, letting ℝ_≥0_ denote the set of non-negative reals and *AP* be a finite set of atomic propositions used to label states with properties of interest, a CTMC is a tuple (*S*,*R*,*L*) where:

• *S* is a finite set of states;

• *R* : (*S* × *S*) *→* ℝ_≥0_ is the transition rate matrix, which assigns rates to each pair of states;

• *L* : *S* → 2^AP^ is a labelling function which associates each state with a set of atomic propositions.

The probability of a transition between states *s* and *s′* being triggered within *t* time-units is 1 – *e*^–*R*(*s*,*s′*).*t*^. The time spent in state *s* before any such transition occurs is exponentially distributed with the rate *E*(*s*) = ∑_*s′*∈*S *_*R*(*s*, *s′*), called the *exit rate*. The probability of moving to state *s′* is given by *R*(*s*, *s′*)*/E*(*s*). In this work we have used PRISM tool to describe and analyze our biological model. PRISM is a known probabilistic model checker that provides support for CTMC models. The properties in PRISM should be specified using the Continuos Stochastic Logic (CSL) [5], which is based on the Computational Tree Logic (CTL) and the Probabilistic Computation Tree Logic (PCTL). The syntax of CSL formulas is the following:

where *a* ranges over a set of atomic propositions, *p* ∈ [0, 1], ⊴ ∈ {>, <, ≥, ≤} and *I* is an interval of ℝ_≥0_. There are two types of CSL properties: transient  and steady-state . For this current work we are only interested in transient or time-dependent properties. A formula  is true in state *s* if the probability that *ϕ* is satisfied by the paths starting from state *s* meets the bound ⊴*p*. Path formulas are constructed using the **X** (next) operator and the **U***^I^* (time-bounded until) operator. The path formula **X**Φ is true if Φ is satisfied in the next state, whereas Φ_1 _**U***^I^* Φ_2_ is true if Φ_2_ is held at some time instant in the interval *I* and at all preceding time instants Φ_1_ holds. Other operators can be derived from this minimal set of CSL operators. Two of them, which will be used in this work, are the *eventually* operator **F***^I^* Φ, which is true if Φ is satisfied in some time instant in the interval I, and the *always* operator **G***^I^* Φ, which is true if Φ is satisfied in every time instant in the interval *I*. It is worth to note that the interval *I* can be omitted in the operators **U**, **F** and **G** which means that the interval is [0, ∞].

Furthermore, PRISM lets a CTMC be augmented with **rewards,** which are structures that associate real values with states or transitions. The state-rewards are accumulated in proportion to the time spent in the state, whereas the transition-rewards are accumulated each time the transition is taken. In PRISM, these are described using the

**rewards** ”*rewardname*” …**endrewards**

construct and are specified using multiple reward items of the form

to describe state-rewards and transition-rewards, respectively. In the previous definition, *g* is a predicate, *a* is a label for a set of commands that represent a transition in the system and *r* is a real-valued expression, which can contain variables and constants from the model. A state-reward item assigns the real value, resulting from the evaluation of *r* expression, to all states satisfying the predicate *g* and a transition-reward item assigns the real value to all transitions labelled with *a* and from states satisfying *g*.

Given the definition of the reward items, some properties can be used to recover amounts related to them. For example, the property ”what is the expected number of reactions between species A and B before a reaction between species A and C happens?” can only be asked with the reward mechanism. Two of the property types related to rewards which will be used in this work are  and . The former property is true, from a state *s*, if the expected state-reward at time instant *t* meets the bound ⊴*r*, whereas the later property is true, from a state *s*, if the reward accumulated along a path until a point where Φ is true meets the bound ⊴*r*. The total reward for a path in a CTMC is the sum of the state-rewards along the path plus the sum of the transition-rewards for each transition between these states. The state-reward assigned to each state of the model is interpreted as the rate at which rewards are accumulated in that state, i.e. if *t* time units are spent in a state with state-reward *r*, the accumulated reward in that state is *r* × *t*. The bounds ⊴*p* and ⊴*r* may not be specified, in which case the probability or reward is calculated in PRISM.

#### PRISM algorithm

The techniques that are implemented in PRISM to solve the PMC problem for CTMC models with rewards include *graph-theoretical algorithms* and *numerical computation*. The former operates on the underlying graph structure of a markov chain to determine, for example, the set of reachable states in a model, or to check qualitative properties. The latter is required for the calculation of probabilities and reward values. While iterative methods to solve linear equation systems are used for answering question related to the steady-state behavior of the model, i.e. its behavior in the long-run or equilibrium, uniformisation is used for transient probability computation. Moreover, PRISM uses a structure called *multi-terminal binary decision diagrams* (MTBDD) for representing compactly the graph structure of a markov chain. More details about PRISM engineering can be found in [12].

### Sodium-potassium pump specification

#### Discrete chemistry

The entities in our model are ion species (, , , ), molecules (ATP, P_i_, ADP) and the Na,K-ATPase (the pump enzyme) which can interact through six elementary reactions (see Fig. 2). In this work the amount of each representative of these species is modeled as a discrete quantity, not using a continuous function. Then, we have converted the amount of initial representatives of species from molarity (M) as shown in Table 1 to counts of molecules and ions. As some rates are also defined in terms of molarity, we have also converted them into stochastic rates  and , which regard counts of molecules and ions.

In order to convert the initial amount of molecules and ions given in molarity ([*X*]) into counts of molecules and ions (*#X*), we have used the following biological definition:(1)

where *V* is the cell volume and *N*_A_ is the Avogadro constant.

Moreover, in order to convert the rates from continuous chemistry to discrete chemistry we have used the Gillespie’s conversion [1]:(2)

where *κ* is the molecularity of the reaction. Molecularity in chemistry is the number of entities that are involved in a reaction. For example, in the simple reaction *A* + 2*B* → *AB*_2_, the reagents are *A* and 2*B* and the *κ* value is 1 + 2 = 3. The  rates are obtained in the same way as the  rates.

#### Law of mass action

The law of mass action states that a reaction rate is proportional to the concentration of its reagents. Then, we have to take into account the reagent concentrations in our model. Regarding the discrete chemistry conversion discussed in last subsection and the fourth step in the Na,K-pump cycle (see Fig. 2)(3)

the final rate *r*_4_ is given as follows:(4)

Given a reagent species, we have to raise its concentration to its molecularity. The final rates for the other sodium-pump mechanism steps are obtained similarly, see [8] for details.

#### PRISM specification

We now illustrate how to specify our Na,K-pump model in the PRISM language. Part of the model is presented in *PRISM Model* code. The complete model is available in [13]. A CTMC description in PRISM language should start with the keyword **ctmc** and comprises a set of *modules*, whose states are represented by a set of finite-ranging *variables*. In our model, there is a module for each species involved in the Na,K-pump: **na**, **k**, **atp**, **adp**, **p** and the **pump** enzyme. There are also two finite-ranging variables in k module: *kIn* and *kOut* that describe, respectively, the number of potassium ions inside and outside the cell. On the other hand, there are six variables in the module **pump** and each of them represents one possible enzyme state, according with the cycle in Fig. 2.

The behavior of each module, i.e. the changes in states which it can undergo, is specified by a number of *guarded commands* of the form []*g* → *r* : *u*. The interpretation of a command is that if the predicate (guard) *g* is true, then the system is updated according to *u*, which comprises one or more statements of the form (*x′* = …) indicating how the value of variable *x* is changed. The rate at which this occurs is given by *r*, i.e. this is the value that will be attached to the corresponding transition in the underlying CTMC. PRISM also supports *synchronization* between modules in the style of process algebras. This is achieved by labelling commands with *actions* (placed between the initial square brackets).

Transitions in different modules labelled with the same action occur simultaneously. The rate of synchronized transitions is equal to the product of the individual rates of the commands of the different modules that synchronize. In our model, the required updates when the fourth pump reaction (given in (3)) happens are represented by the commands labelled with action **r4**. Then, there is a decrease in the number of potassium ions outside the cell and the pump changes its state. The final rate when this reaction happens is *r*4*rate* ∗ *E2P* ∗ *pow*(*kOut*, 2), where *r*4*rate* denotes the  stochastic rate derived from the kinetic rates using the calculation described in Sect. *Methods - Sodium-potassium pump specification -Discrete chemistry*, *E2P* denotes the amount of pumps in *E2P* state and *pow*(*kOut*,2) ≡ *kOut*^2^. We can extend our existing model by allowing more than one pump to occur in the system using the **NP** constant, which can assume any integer value to represent the number of pumps.

Finally, we add reward structures to our model as shown in *Rewards to PRISM Model*. We have defined two rewards: **kOut** and **time**. The former assigns the current number of potassium ions outside the cell to every state in the system. This can be used to compute the expected amount of potassium ions outside the cell in a specific time for example. The latter simply assigns a state-reward of 1 to all states in the model and it is useful to analyze the total expected time before an event occurs.

## Results and discussion

In the following analysis, all properties have been obtained considering a model with only one pump. Moreover, Table 2 shows that as the cell volume grows, so will be size of the model and the time required to build and check it. We have used a cell volume equal to 10^–20^ liters for analysis in the following sections. This type of abstraction strategy is common for modeling biological systems as discussed in [14].

**Table 2:** Model size ranging the cell volume.

Cell Volume(l)	#States	#Transitions	Time to Build	Time to Check
10^–22^	9	16	0.03 sec	2 min 45.54 sec
10^–21^	32	62	0.29 sec	51.94 sec
10^–20^	194	386	47.45 sec	4 min 45.35 sec
10^–19^	1 838	3 674	1 h 48 min 29.03 sec	1 h 2 min 18.98 sec
10^–18^	?	?	> 7 days	?

Model size (*States and Transitions*) and *Time to Build* the model and *Time to Check* the property *R*{”*kOut*”} =?[*I* = 5], which means the expected potassium outside the cell at the time equal to 5 seconds. In the experiments, the number of pumps in the model is one. The machine that we have used to run the experiments is an Intel(R) Xeon(R) CPU X3323, 2.50GHz and has 17 GB of RAM memory. The ”?” symbol means that the value has not been determined so far, because of the long execution time.

### Discovering rare events

Uncommon events can have a significant impact in any system and particularly in biological systems. For example, if a particular combination of reagents can cause a pump to block permanently, it can cause cell death. No matter how unlikely this event is, if it happens the consequences are critical. Traditional analysis methods such as stochastic simulations can miss uncommon or rare events, because they simulate random paths in the evolution of the system, and if the event is rare, it is not likely that it will be simulated in a viable amount of time. PMC, however, can identify these events by looking for them. By stating a property that is true if such an event occurs, PMC can identify the conditions for its occurrence, and as a consequence, uncover hidden but potentially important behaviors in the system.

**Figure 3:**
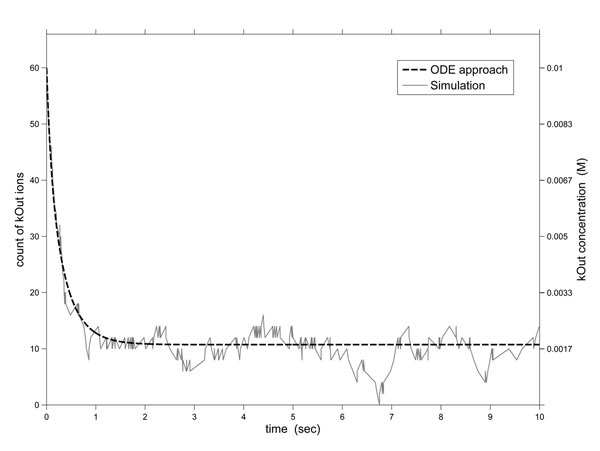
**Traditional solutions for potassium outside the cell in the sodium-potassium pump model.** Potassium concentration in M outside the cell over time for the ODE approach (dashed line and y axis on the right) and count of potassium ions outside the cell given by a simulation trace (solid line and y axis on the left).

Our first analysis shows how model checking can be used to identify rare events in the Na,K-pump. Figure 3 presents the potassium concentration in *M* outside the cell over time for the ODE approach (dashed line and *y* axis on the right), which uses a deterministic and continuous pump model. The model was built and solved in MATLAB as described in [15]. The figure also presents the count of potassium ions outside the cell given by a simulation trace (solid line and *y* axis on the left) of the discrete and stochastic pump model. The simulation trace was obtained using the BIONETGEN tool [16], which provides an implementation of the direct method of Gillespie.

As can be seen in the ODE approach, the potassium outside the cell is decreasing until around 2 seconds, and its concentration converges on about 0.0018 M. However, this average behavior hides some important traces, as it is shown in the same figure, where the potassium count outside the cell, after the fast decrease until about 2 time units, will oscillate around 12 and might end, i.e. it can eventually reach value 0. However, the probability of this event *potassium outside the cell ends* is extremely low (6.33 × 10^–3^) during the first 10 seconds. This probability value was determined using the CSL property *P* =? [*F* <= 10 *kOut* = 0]. Notice that there is a significant difference between a rare event and an impossible event. If the event is rare we may decide, based on its occurrence probability, to investigate it further or not. If it is an important event that may cause death it may merit further studies, whereas if it *cannot* happen, we need not worry about it. Properties such as these can help direct future researches and shorten scientific discovery times.

Thus, whereas a deterministic simulation *will* not identify this rare event *potassium outside the cell ends* because it captures only average behaviors, the stochastic simulation approach *may* not capture traces where it happens, depending on the simulation time and on the number of simulated traces.

However PMC can provide stochastic simulation with some hints in this sense. As it lets us know in advance that the rare event happens with probability equal to 6.33 × 10^–3^ in the first 10 seconds, if the stochastic simulation time being considered is 10, in a sample of 1000 traces, for example, about 6 or 7 of them will probably show the rare event.

It is also important to note that in PMC the time is continuous, while in stochastic simulation it is discrete. Hence, if the duration of an event of interest is smaller than the time step being considered in the simulation, it will not be captured, whereas it will be considered in the PMC model. As will be shown, PMC can give some clues for stochastic simulation in order to address these issues. The CSL property shown below (followed by the model checker answer)(5)

ensures that in all model traces the potassium outside the cell, in fact, will eventually end. Additionally, in order to know about the expected time for this event to happen, properties (6) and (7) can be used:(6)(7)

Property (6) means *What is the expected time to the potassium outside the cell being over?*. The model checker answer was 1287 seconds. The reward structure for this property associates reward 1 with each state (see the reward structure ”time” in Sect. *Methods - Sodium-potassium pump specification - PRISM Specification*). On the other hand, property (7) asks *about the probability of potassium outside the cell eventually being over in the first 1287 seconds*, whose answer is 0.63. Thus, we can conclude that during the first 1287 seconds the potassium outside the cell ends in around 63 % of the traces of the system. In this case the expected time is very long and unlikely to be reached before another event occurs. However, the same technique can be used to model reaction time to the presence of a toxin, for example, and the expected time can be crucial to the survival of the cell.

Finally, the following properties are used to reason about the *maximum and minimum expected time for the system to go from a state where potassium outside the cell is over to a state where this species is not more over*:(8)(9)

There is more than one state where potassium outside the cell is over and, therefore, **min** and **max** are used to return the minimum and maximum expected time to reach a state where *kOut* > 0, ranging over all start states that satisfy *kOut* = 0. The model checking answers for (8) and (9) properties are, respectively, 111 milliseconds and 14 milliseconds. This minimum expected time for the event duration can be used as a guideline for choosing the time step in stochastic simulation to guarantee that no such events will be lost. Another event that can be easily identified with model checking is if *after that potassium outside the cell is ended*, *its amount will eventually return to the initial count KO*. Property (10) is used to verify if this event happens in all traces in the model, whereas property (11) is used to determine the expected time for the event to happen:(10)(11)

where ”kOutOver” is a label to *kOut* = 0. The model checker result for property (10) was true and the expected time computed in property (11) is about 132, 515 seconds.

Thus, two significant events in the system *potassium outside the cell will end and the potassium outside the cell will return to its initial count* will happen in all traces of the pump model and lead us to study about their recurrence in the long term.

### Reversibility of the sodium-potassium pump

Due to the fact that there are backwards and forwards transitions for all reactions involved in the Na,K-pump mechanism, as is shown in Fig. 2, and that many of these transition rates depend on transmembrane substrates, the pump mechanism is automatically reversible, i.e the reactions can be run either forwards or in reverse direction, depending on changes in the amount of substrates. Thus, given the initial concentrations of the substrates, we can consider that the pump performs two main steps. First it runs following the forward reactions and reaches the configuration where substrates reach a maximum or minimum concentration. Then, given these changes in the amount of substrates, the pump returns to the initial configuration through the reverse reactions. Of course, it is possible that the reverse reactions can be followed during the first step, and, similarly, forward reactions can be followed during the second step. In the forward running, Na,K-pump uses one ATP to perform the electrogenic exchange of 2 potassium ions from outside to inside the cell in exchange for 3 sodium ions from inside to outside the cell. In the reverse direction ATP can be produced from ADP and P_i_.

Without loss of generality, we will study this reversible pump behavior in terms of the potassium amount outside the cell, which will be the species under observation. We can see this pump reversibility as an infinite oscillation between two values, the initial amount of potassium outside the cell, *KO*, and the final amount of substrates, after the forward running is complete and before the reverse running starts. In this final configuration, the potassium outside the cell ends, i.e. it reaches its minimum value (*kOut* = 0). This pump reversibility is expressed through CSL property (12), which means *What is the probability that kOut oscillation between 0 and KO values will never terminate?*(12)

where *i* is *KO* and *j* is 0. The result of CSL property (12) is 1, which proves that the events *potassium outside the cell ends* and *potassium outside the cell reaches the initial amount* happen infinitely often, i.e. the automatic pump reversibility property of the pump is true. This pump property can not be seen in the Fig. 3, because the study of the average behavior of the pump overlooks some aspects of its reversibility. Moreover we are reasoning about the pump infinite behavior, which cannot be achieved through generating and analysis of finite-time trajectories with stochastic simulation. Property (12) can be used to check if the concentration of some species oscillates between any two values *i* and *j*.

### Understanding the pump cycle

In this section we present a study of the Na,K-pump mechanism in terms of the rates in the cycle shown in Fig. 2, in order to understand why the depletion of potassium outside the cell and consequently the pump reversibility can take long periods of time to be completed.

We now introduce some definitions and extensions in the previous PRISM pump model that will be used later. First, we compute the positive or ascending trend [17] of a transition rate *r*(*s*, *s_i_*) from a current state *s*:

where *E*(*s*) is the exit rate of state *s*, i.e., *E*(*s*) = *∑_s′∈S_ r*(*s*, *s′*), being *S* a finite set of states, and *ξ* is a threshold that indicates a positive trend. We have chosen the value 0.6 for *ξ* in our analysis and, therefore, informally an ascending trend for a transition rate *r*(*s*, *s_i_*) means that the probability of the system goes from *s* to *s_i_* (at least 0.6) is greater than goes to any other state *s_j_* (*j* ≠ *i*), whose transition rate *r*(*s*, *s_j_*) > 0.

Thus, we add trend formulas to the previous PRISM model for all transition rates using PRISM resources (*labels* and *formulas*). The code in *PRISM Model Extension 2* illustrates the procedure for computing the positive trend label for the transition rate *r*_1_.

The rate transition *r*_1_ is computed by the formula **rate_r1** and it is different to 0 when the current pump state is *E*_1_. ATP and there is enough sodium inside the cell . In this case, the final value for *r*_1_ is determined in the same way as described in Sect. *Methods - Sodium-potassium pump specification - PRISM Specification*. The other rates are computed in similar way than *r*_1_ and the formula exit rate represents their summation. The probability that *r*_1_ is taken in the current state is given by formula **rate_r1_d**, whereas the label **trend_r1_up** represents if *r*_1_ really has an ascending trend, i.e. ↑ *r*_1_ is 1. Now, we can use the CSL property (13) to identify the rates that never have a positive trend during the system evolution and, consequently those rates that always have an ascending trend:(13)

In our pump model, [*r*] is used to form the trend label of the backward rates, and *i* ranges from 1 up 6, because there are six reactions in the pump system, considering each direction (forward or backward). Thus, the trend ”trend_r1” represents the trend label for the first pump reaction in the forward direction (see Fig. 2), whereas the trend ”trend_rr1” refers to the trend label for the first pump reaction in the backward direction.

**Figure 4:**
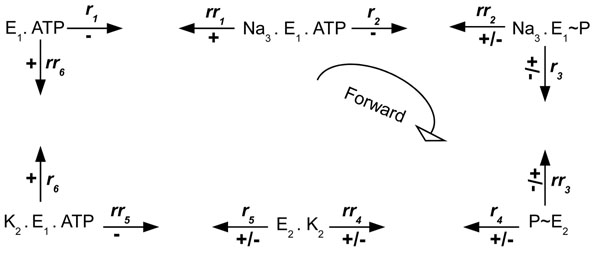
**Summarization of the rate trends in sodium-potassium pump.** Summarization of the trend for all transition rates in the pump cycle presented in Fig. 2. The arrows leaving the pump states are labelled with the rates for the transition between the current state and the following state, which is given by the direction of the arrow. Associated with each arrow, there is also a sign that indicates if the transition rate has always a positive trend (+) or a negative trend (-), and, finally, if the trend can be negative and also positive during the system evolution.

The results for property (13) are summarized in Fig. 4, which shows the trend for all transition rates in the pump cycle presented in Fig. 2. The arrows leaving the pump states (such as *E*_1_.ATP) are labelled with the rates for the transition between the current state and the next state, which is given by the direction of the arrow. Associated with each arrow, there is also a sign that indicates if the transition rate has always a positive trend (+) or a negative trend (-), and, finally, if the trend can be negative and positive during the system evolution (+/-).

We can see that the forward rates *r*_1_ and *r*_2_ always have a negative trend, while *r*_6_ always has a positive trend during the system evolution. Moreover, the trends for the forward rates *r*_3_, *r*_4_ and *r*_5_ can be positive or negative, depending on the changes in the amount of substrates during the pump evolution.

In order to identify the moment when these forward transition rates which don’t have only a positive or negative trend during the system evolution change their trends, we have again extended our PRISM model with the following transition-rewards

In the *PRISM Model Extension 3*, **plusKout** is a reward that assigns 1 to each transition from the state K_2_.*E*_2_ to state *E*_2_ ~ P, which results in the releasing of two potassium ions outside the cell. On the other hand **minusKout** is a reward that assigns 1 to each transition from the state *E*_2_ ~ P to the state K_2_.*E*_2_, which results in the consumption of two potassium ions outside the cell. CSL property (14) determines *the expected count of potassium outside the cell when the rate r*[*r*]*_i_ starts to have a positive trend*:(14)

Using property (14), we can see that *r*_3_, *r*_4_ and *r*_5_ (forward rates) start to have a negative trend only when the potassium outside the cell is, respectively, 21, 7 and 7 (the initial amount of potassium outside the cell in our model is 61).

**Figure 5:**
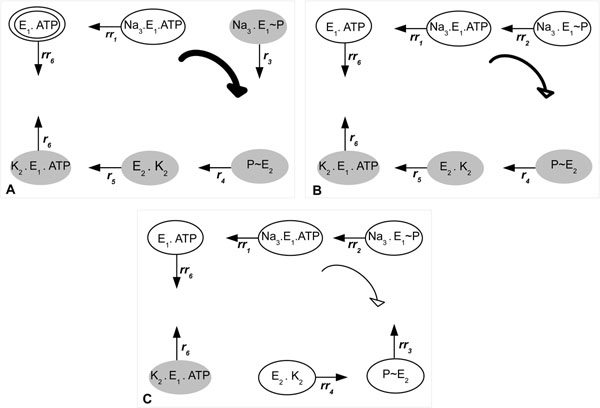
**The main steps of the sodium-potassium pump system in terms of rate trends.** Steps of the sodium-potassium pump determined by the transition rate trends. There are arrows only for the rates that exhibit positive trends. Pump states in gray are those whose the rate in the forward direction has positive trend, whereas those in white have the backward rate exhibiting a positive trend. The thickness of the central arrow in the forward direction indicates the strength to the general trend of the pump in this direction. Thus, the thicker the arrow, the greater the tendency of the pump to run in the forward direction. (A) Initial step. The double circle represents the initial state of the system. (B) Intermediate step. (C) Final step.

Thus, we can divide the pump operation into three main steps, as is shown in Fig. 5. Initially (A), despite the general trend to move backwards due to the positive trends of the backward rates *rr*_1_ and *rr*_6_, once *r*_3_ is taken, the system might complete easily the cycle in the forward direction, because the forward rates *r*_3_, *r*_4_, *r*_5_ and *r*_6_ have a positive trend in the most of the time. The backward rate *rr*_2_ needs that the pump goes in the forward direction awhile, increasing the amount of ADP inside the cell, in order to exhibit a positive trend. When potassium outside the cell reaches the value 21, the rates *r*_3_ and *rr*_2_ changes their trends, starting the intermediate step (B). In this step, the pump can still move in forward direction. The last step (C), starts when the potassium outside the cell reaches the value 7, causing changes in the trends of the forward rates *r*_4_ and *r*_5_. First, rate *r*_4_ no longer has a positive trend, while the negative trend of the backward rate *rr*_3_ is replaced by a positive one. This happens due to the increase of sodium outside the cell, which gives strength to *rr*_3_, and the decrease of potassium outside the cell, which weakens *r*_4_. Second, the forward rate *r*_5_ also stops exhibiting a positive trend, whereas the trend of the backward rate *rr*_4_ starts to be ascending. This change is caused by the accumulation of P_i_ inside the cell and the reduction of ATP due to the pump movement in the forward direction. In step (C) there is a low probability, although is not impossible, that the pump continues its operation in the forward direction, given that the only forward rate with positive trend is *r*_6_, delaying the depletion of potassium outside the cell. In fact, there is a strong general trend for the pump to move backwards, returning to the intermediate step, where the system stays most of the time. Additionally, the pump can move backwards from the intermediate step, returning to the initial configuration. However, this takes long periods of time, given that it is necessary to move against the positive trends of the forward rates *r*_3_, *r*_4_, *r*_5_ and *r*_6_ in the initial step.

As shown in the previous sections, the depletion of potassium outside the cell and the pump reversibility are events that can happen in the pump model. However, they can take longs periods of time to be completed. So the study of this section is important to indicate the reasons for this delay. For example, it is possible to see that the first obstacle in the normal operation of the pump is the accumulation of ADP inside the cell which causes the reversion of the *r*_3_ trend. This may indicate a specific aspect of the system that merits further studies. This result may lead to a more precise study because it tells us in detail what has happened (accumulation of ADP inside the cell) and not simply that the pump has reversed its behavior. Results such as these can uncover important hidden behaviors that can speed up further experiments and increase their accuracy.

### Validation of the PRISM model

In this section we will show that our PRISM model can produce similar results when compared to the stochastic and deterministic simulations. Property (15) allows us to know the expected amount of potassium outside the cell in time *T*, which specified in the property:(15)

**Figure 6:**
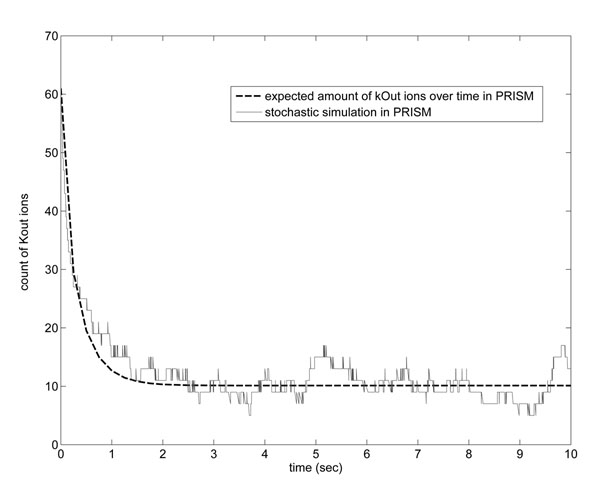
**PRISM curves for the sodium-potassium pump model.** Expected amount of potassium outside the cell over time (dashed line) given by property (15) and count of potassium ions outside the cell given by a PRISM simulation trace (solid line).

The label ”kOut” is a reward name defined as shown in *Rewards to PRISM Model, Sect. Methods - Sodium-potassium pump specification - Prism specification*. PRISM supports *experiments*, which is a way of automating multiple instances of model checking. In our case, this is done by ranging the constant *T* from, for example, 0 up to 10, with steps of 0.25. The resulting graph is shown in Fig. 6 (dashed line), which is very similar to deterministic curve shown in Fig. 3. We also got a similar trajectory using the PRISM tool, Fig. 6 (solid line), which besides verification can also perform stochastic simulation of the model that mimics the Gillespie method. Thus, we can see that PRISM results for the sodium-potassium pump are very close to those obtained using the traditional approaches.

## Conclusions

In this work we use a stochastic modeling approach and *probabilistic model checker* to model and analyze the Na,K-pump which provides a new perspective on the study of the behavior of this system. It inherits many of the advantages of model checking, including the use of a formal specification of the system and the fact that the approach is exhaustive, analyzing all possible behaviors of the system.

We have presented a quantitative formal specification of the Na,K-pump, based on a set of elementary reactions. All the process to build the model in the PRISM tool, taking into account a discrete chemistry and the Law of Mass Action has been described. Moreover, we have also checked some rare quantitative properties such as the depletion of sodium potassium outside the cell and the pump reversibility that can be addressed easily using model checking, whereas with the other traditional approaches, such as simulation and ODE methodology, it can be difficult.

Furthermore, using model checking we have shown that these events happen infinitely often. These properties cannot be addressed using simulations, given that they are, by definition, time-finite approaches and, additionally, do not construct the mathematical model which represents all possible states that a system can be.

Moreover, we have used transition rate trends, in order to understand the pump behavior and why it takes a long period of time to express completely the reversibility property.

Finally, we have shown that probabilistic model checking can be used along with other well established approaches to extend the pump behavior knowledge. Then, after we know that the event *potassium outside the cell ends* happens, through model checking, we can focus the other approaches to identify and understand it better.

In practice, the main objective of this work is to provide biologists with hints related to important and interesting events that should be checked in more detail using biological experiments. Thus, biological experiments could be preceded by model checking analysis, which can be used very efficiently, for example, for rejecting impossible hypothesis or for orienting biologists toward logical possible situations. In this way, instead of performing many experiments, the biologists will focus on those that are as pointed out as possible by the mathematical model.

Future works include making our Na,K-pump model more dynamic, adding other actual cell membrane aspects and systems. In order to deal with the large state space, given the big number of ions and molecules, an abstraction of CTMCs based on discrete levels of concentrations, namely CTMC with levels [18], is already in progress.

## Competing interests

The authors declare that they have no competing interests.

## Authors' contributions

MAC and SVAC carried out the model checking studies, analysis and validation of the results. MAC studied the biological system, found the experimental data related to it, built the model in the PRISM tool and created the CLS properties about the biological system. SVAC and ACF participated in the study design and coordination. All authors helped to draft the manuscript and were involved in the read, review and approval of the final manuscript.
